# Supplementary material for: The formation of avian montane diversity across barriers and along elevational gradients
Source: Nat Commun. 2022 Jan 12;13:268. doi: 10.1038/s41467-021-27858-5 (PMC8755808; doi:10.1038/s41467-021-27858-5)
Supplement: Supplementary file 1 — Supplementary figures and tables [file 41467_2021_27858_MOESM1_ESM.docx]

**Supplementary Information for**

**The formation of avian montane diversity across barriers and along elevational gradients**

José Martín Pujolar^1,2^, Mozes P.K. Blom^3,4^, Andrew Hart Reeve^1^, Jonathan D. Kennedy^1^, Petter Zahl Marki^1^, Thorfinn S. Korneliussen^5^, Benjamin G. Freeman^6^, Katerina Sam^7,8^, Ethan Linck^9^, Tri Haryoko^10^, Bulisa Iova^11^, Bonny Koane^12^, Gibson Maiah^12^, Luda Paul^12^, Martin Irestedt^3^, Knud Andreas Jønsson^1^

*^1^Natural History Museum of Denmark, University of Copenhagen, Universitetsparken 15, DK-2100 Copenhagen, Denmark.*

*^2^Centre for Ocean Life, DTU Aqua, Kemitorvet, building 202, DK-2800 Kgs. Lyngby, denmark*

*^3^Department of Bioinformatics and Genetics, Swedish Museum of Natural History, SE-104 05, Stockholm, Sweden.*

*^4^Museum für Naturkunde, Leibniz Institut für Evolutions- und Biodiversitätsforschung, Berlin, Germany.*

*^5^Lundbeck Foundation GeoGenetics Center, Globe Institute, University of Copenhagen, Denmark,*

*^6^Biodiversity Research Centre, University of British Columbia, Vancouver, British Columbia, Canada.*

*^7^Biology Centre of Czech Academy of Sciences, Institute of Entomology, Branisovska 31, Ceske Budejovice, Czech Republic.*

*^8^University of South Bohemia, Faculty of Science, Branisovska 1760, Ceske Budejovice, Czech Republic.*

*^9^Department of Biology & Museum of Southwestern Biology, University of New Mexico, Albuqurque, NM, USA*

*^10^Research Centre for Biology, Indonesian Institute of Sciences (LIPI), Museum Zoologicum Bogoriense, Jl. Raya Jakarta-Bogor Km 46, Cibinong 16911, Indonesia.*

*^11^Papua New Guinea National Museum and Art Gallery, Port Moresby, Papua New Guinea.*

*^12^The New Guinea Binatang Research Centre, Madang, Papua New Guinea.*

*corresponding author Knud Andreas Jønsson

**Email:** [kajonsson@snm.ku.dk](mailto:kajonsson@snm.ku.dk)

**
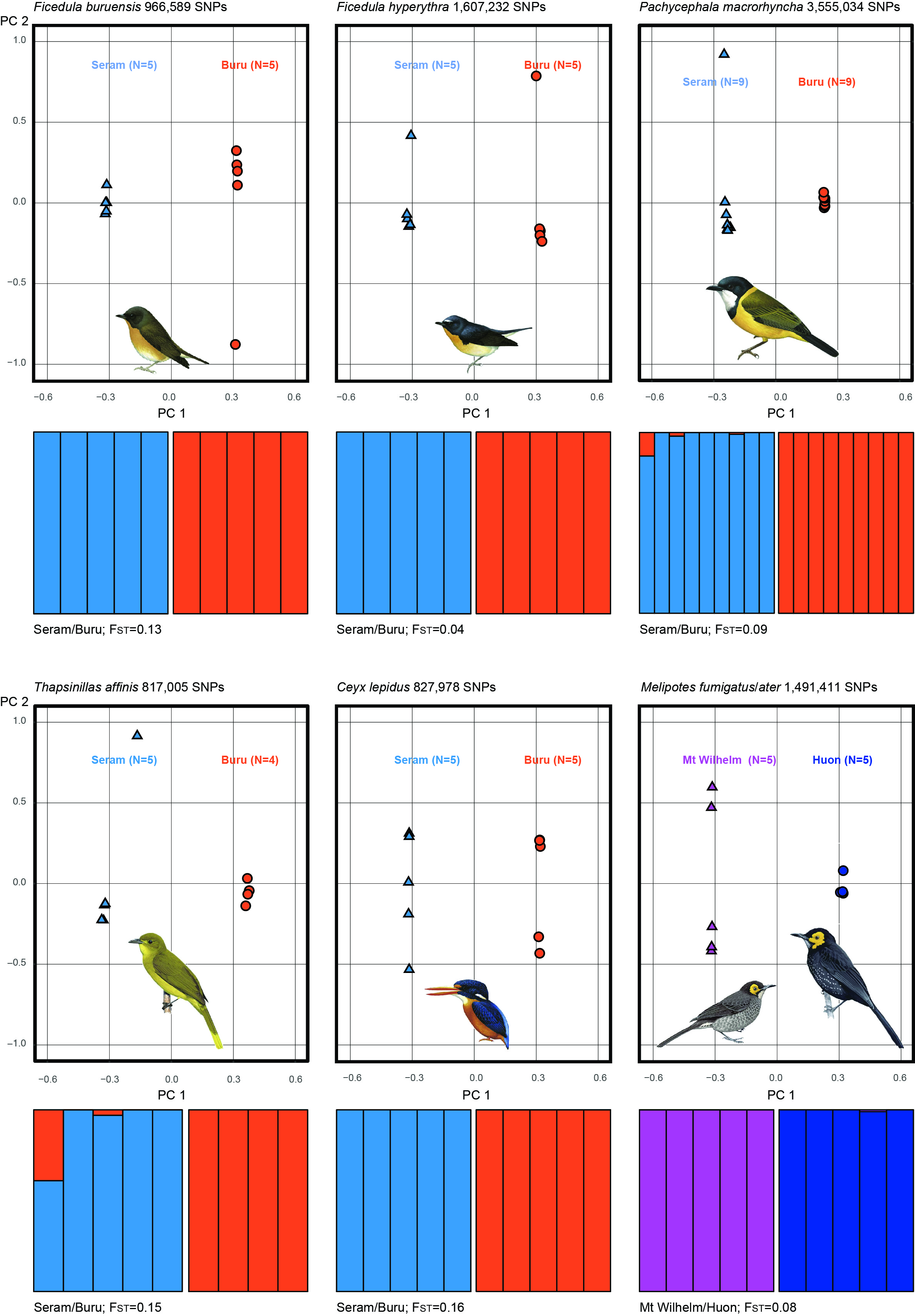
**

**
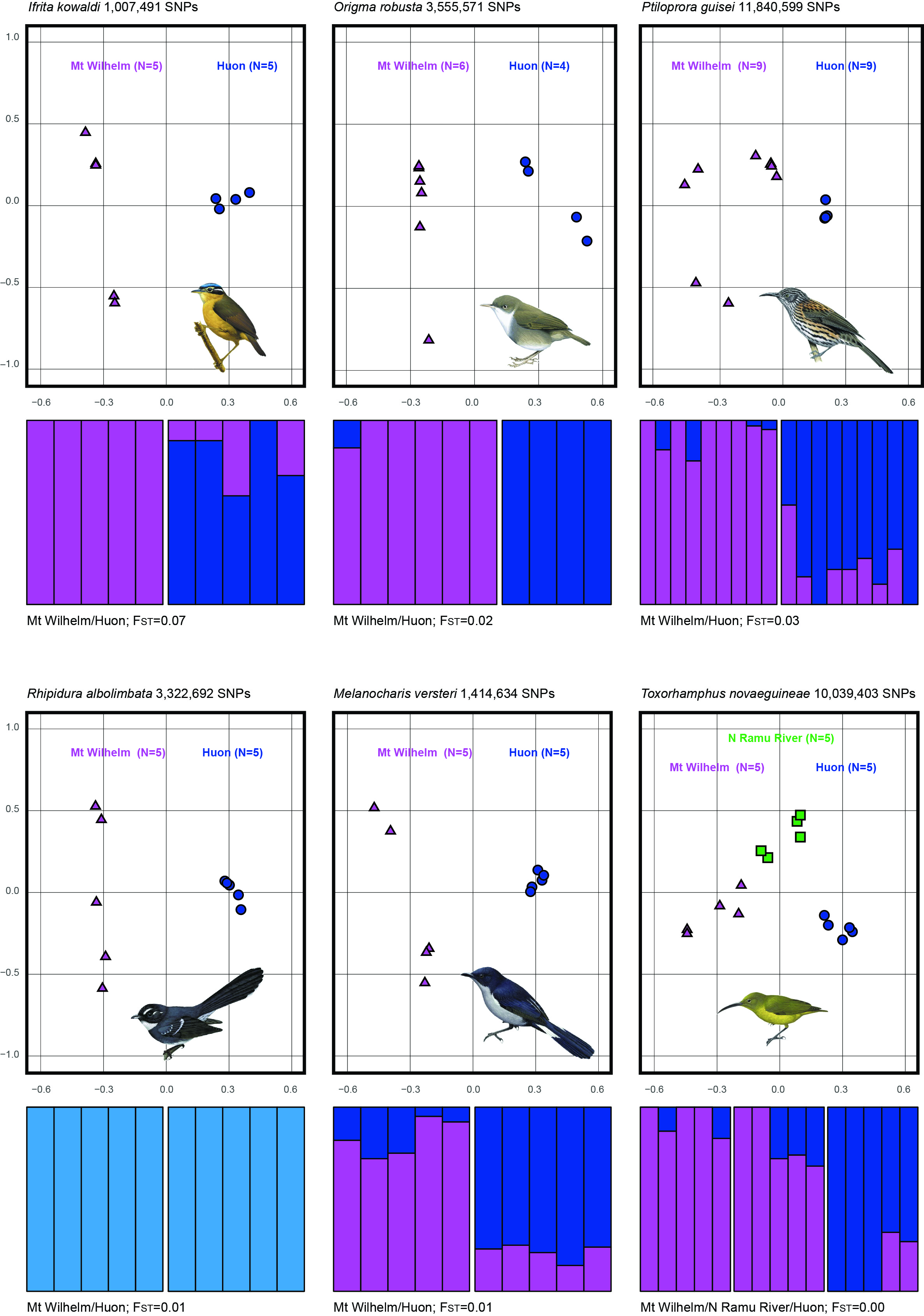
**

**
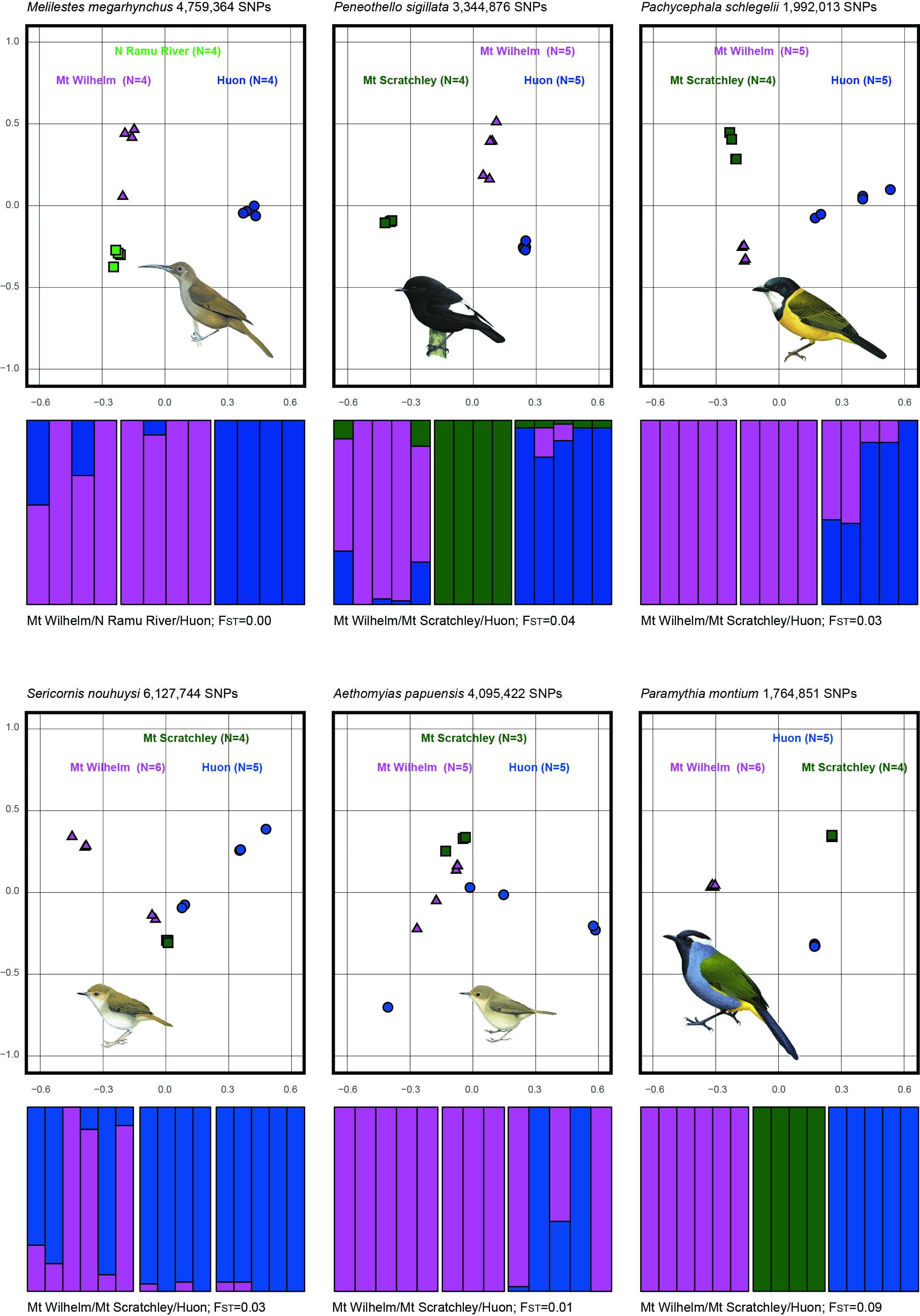
**

**Supplementary Figure 1A, 1B, 1C: PCA and admixture plots.** PCA plots and admixture analysis in STRUCTURE for populations of all 18 species in our study, including (a) Moluccan species (*Ficedula buruensis*, *Ficedula hyperythra*, *Pachycephala macrorhyncha*, *Thapsinillas affinis* and *Ceyx lepidus*) as well as the New Guinean species pair *Melipotes* *fumigatus*/*ater*, (b) New Guinean species (*Ifrita kowaldi*, *Origma robusta*, *Ptiloprora guisei*, *Rhipidura albolimbata*, *Melanocharis versteri* and *Toxorhamphus novaeguineae*) and (c) New Guinean species (*Melilestes megarhynchus*, *Peneothello sigillata*, *Pachycephala schlegelii*, *Sericornis nouhuysi*, *Aethomyias papuensis* and *Paramythia montium*). PC axis 1 explains between 1.13-2.92 % of the variation (mean = 1.82 % median = 1.68%) whereas PC axis 2 explains between 0.85-1.97 % of the variation (mean = 1.17 % median = 1.06%). In STRUCTURE, individuals were assigned on the basis of the most likely K. Admixture proportions are shown for all individuals, each individual represented by a vertical bar. Illustrations of the focal bird species from del Hoyo et al. (1).


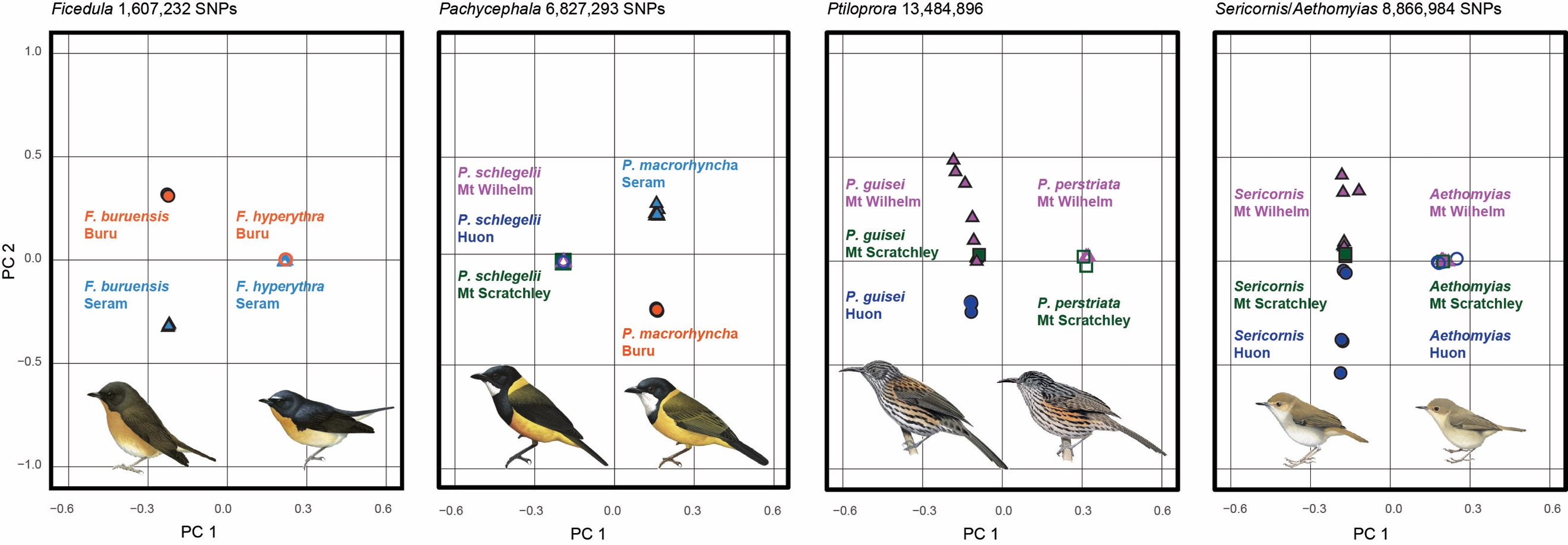


**Supplementary Figure 2: PCA plots of congeneric species.** for all instances of congeneric species in our study including *Ficedula hyperythra* and *F. buruensis*, *Sericornis nouhuysi* and *Aethomyias papuensis* (formerly *Sericornis papuensis*), *Pachycephala schlegelii* and *P. macrorhyncha*, and *Ptiloprora guisei* and *P. perstriata*. Axis 1 explains between 2.85-5.84% of the variation (mean = 4.91%) whereas axis 2 explains between 1.66-3.15% of the variation (mean = 2.20%). Illustrations of the focal bird species from del Hoyo et al. (1).

**
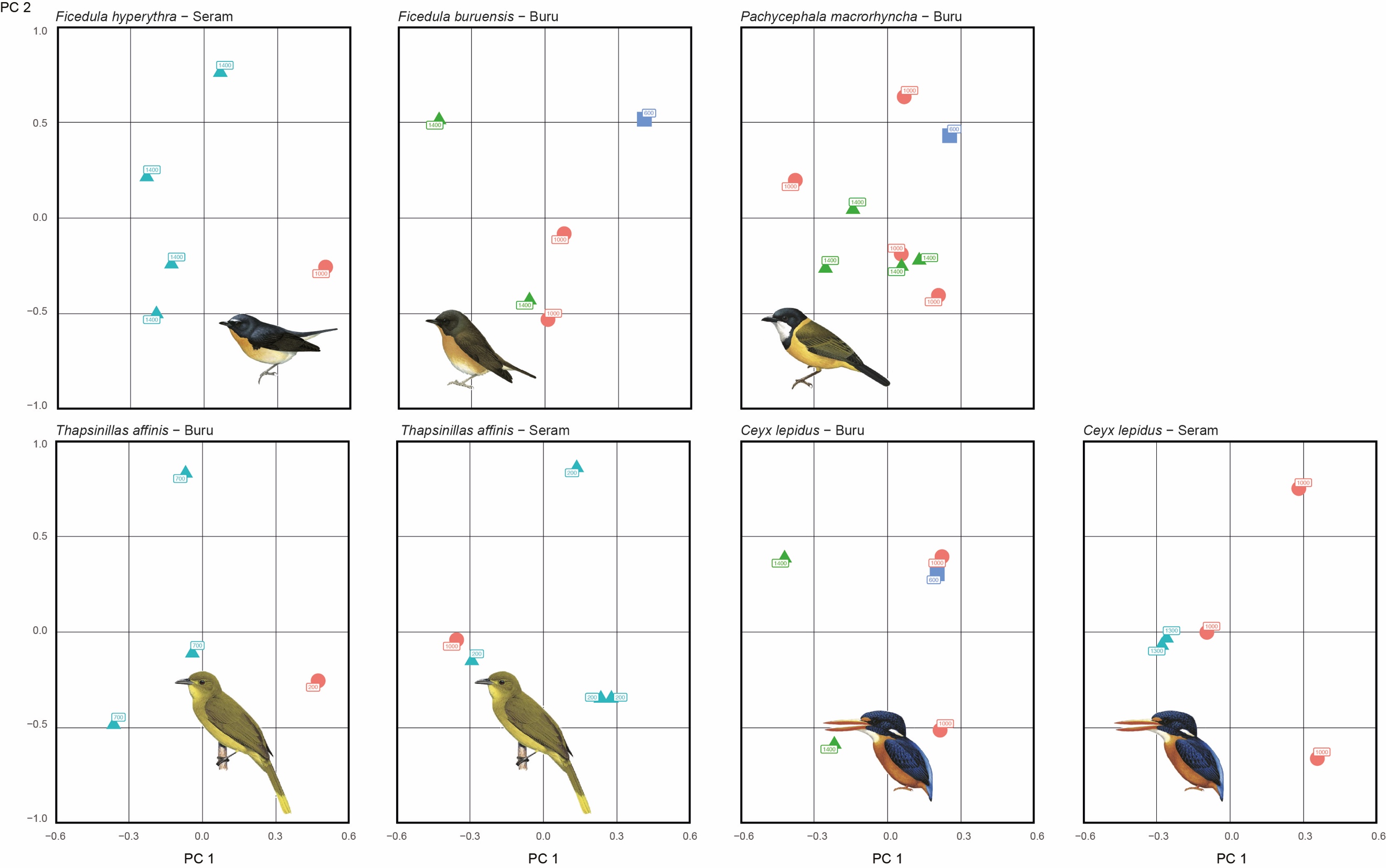
**

**
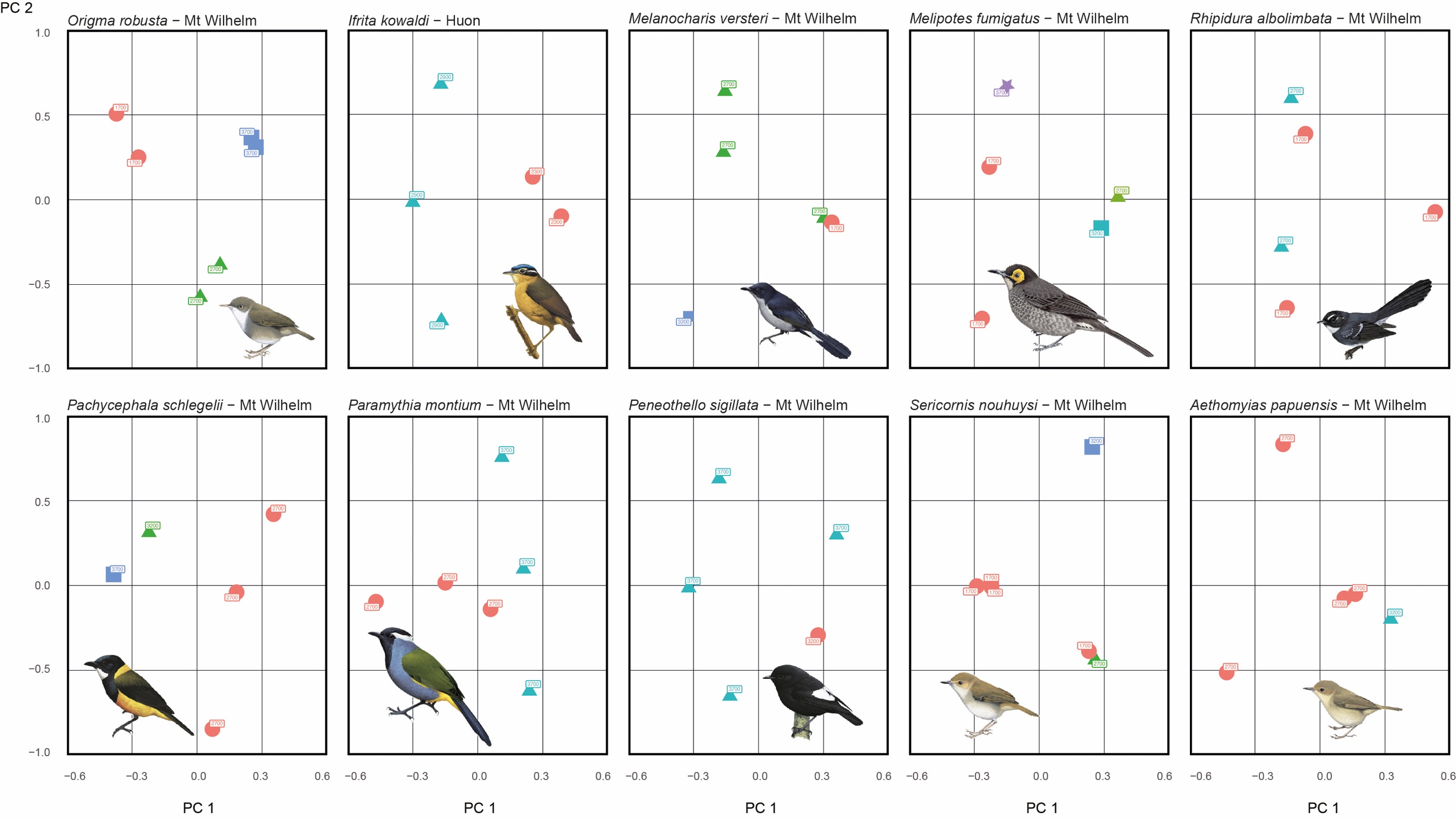
**

**Supplementary Figure 3A, 3B**: **PCA plots from individual gradients.** PCA plots for 18 populations for which multiple elevations were sampled, including (a) eight Moluccan species and (b) ten New Guinean species. PC axis 1 explains between 1.011-1.279 % of the variation (mean = 1.072 % median = 1.032%) whereas PC axis 2 explains between 1.001-1.091 % of the variation (mean = 1.023 % median = 1.007%). Illustrations of the focal bird species from del Hoyo et al. (1).


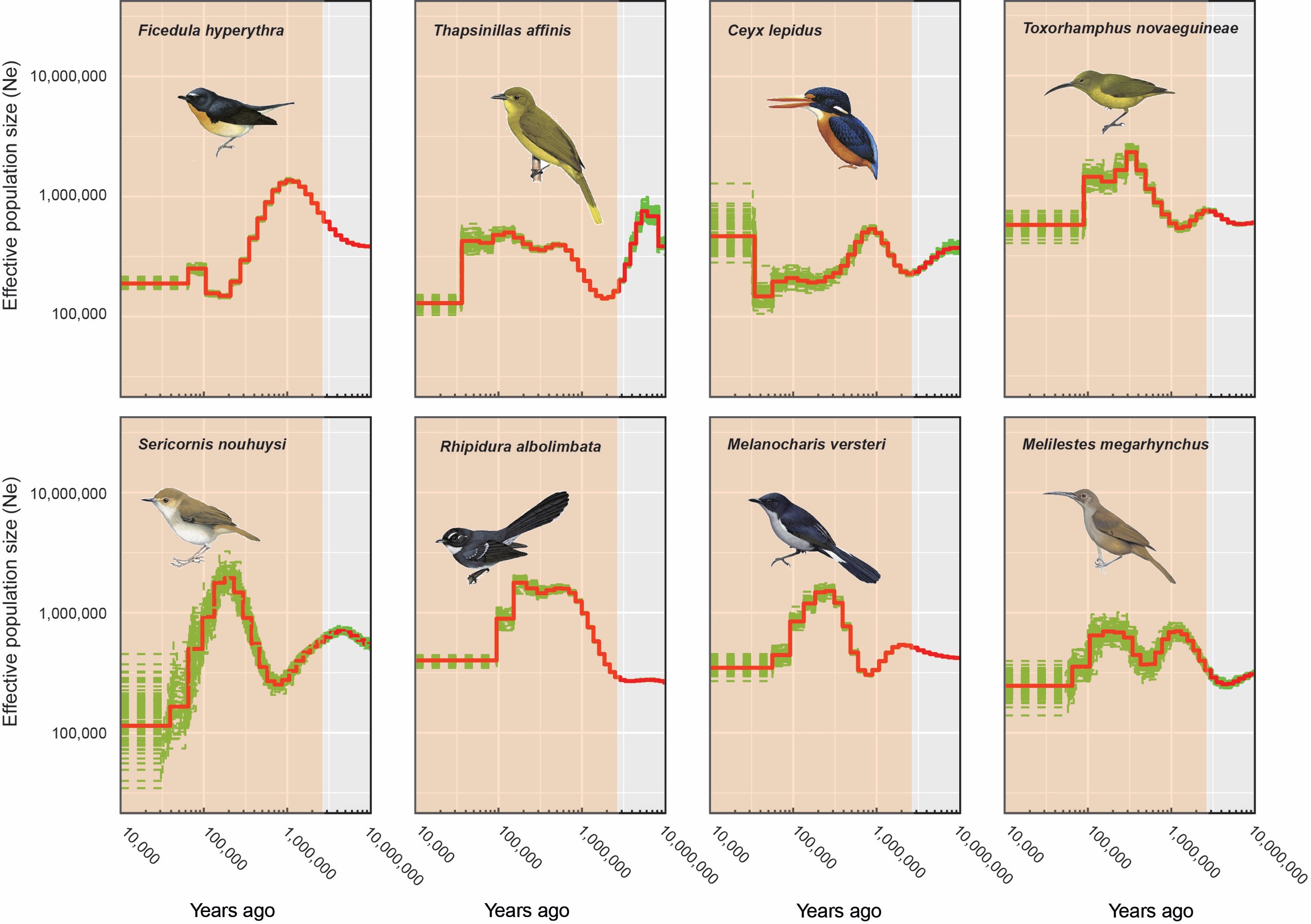


**
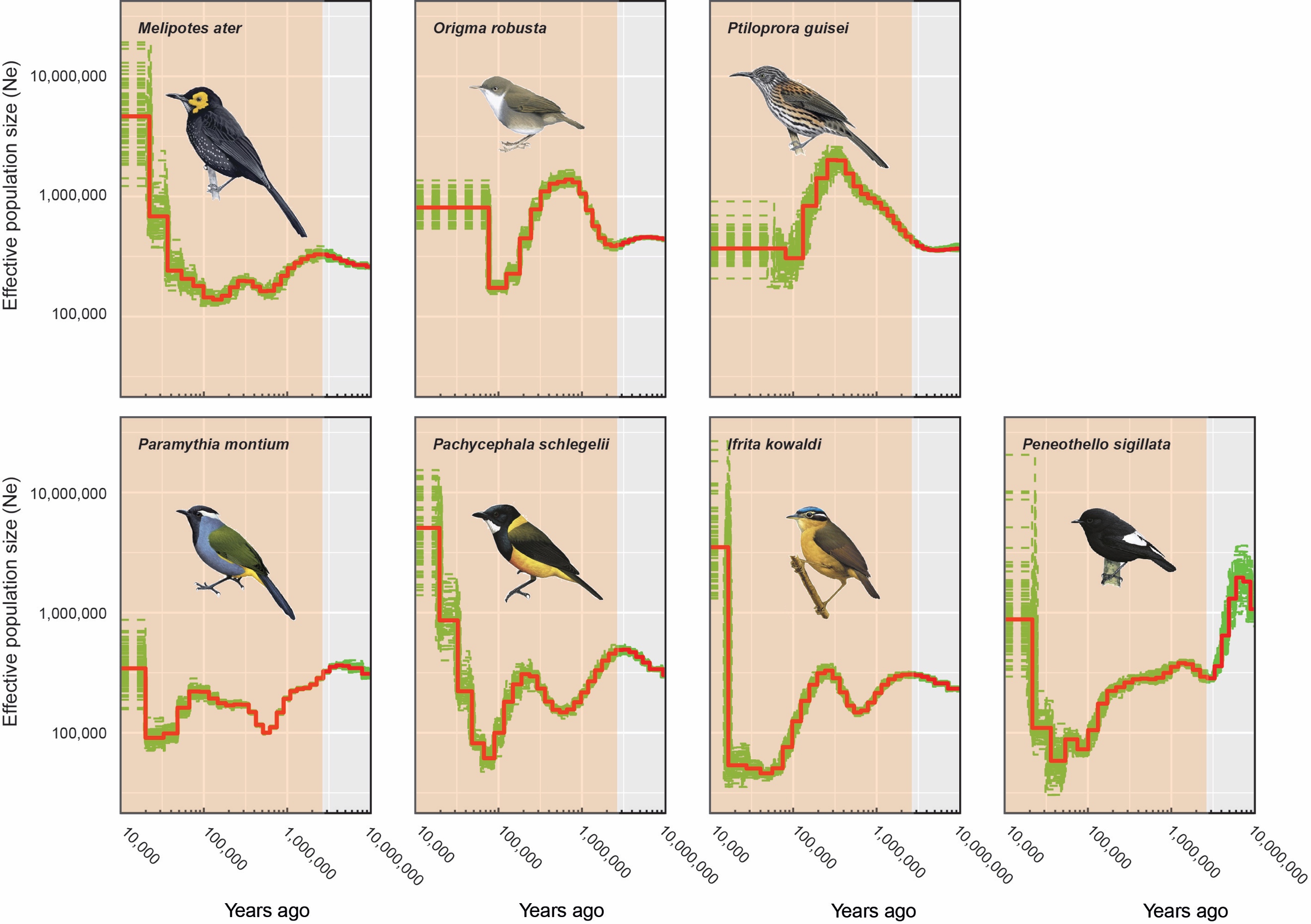
**

**Supplementary Figure 4A, 4B**: **PSMC estimate of the demographic changes (Ne=effective population size) over time** for the bird species in our study including bootstraps. The red curve is the PSMC estimate for the *de novo* sequence data and the green curves indicate PSMC estimates for 100 bootstrapped sequences. Illustrations of the focal bird species from del Hoyo et al. (1).

**
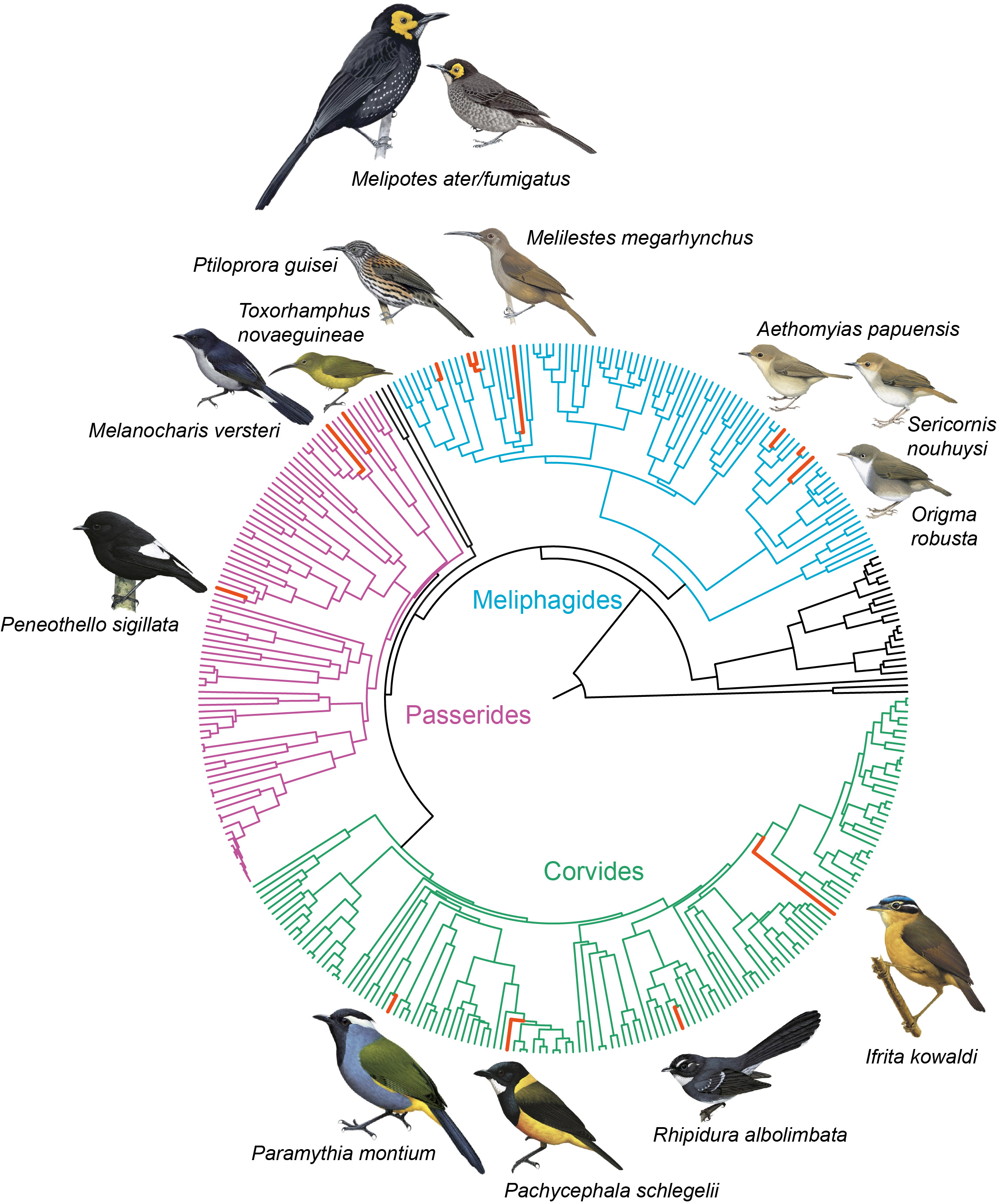
**

**Supplementary Figure 5: The phylogenetic positions of the New Guinean species.** The analysed species herein are shown in a phylogeny including all 336 Passerine bird species of New Guinea based on a concatenated dataset of three mitochondrial genes (ND2, ND3, and cytb) and three nuclear introns (GAPDH, ODC and Myo2) analysed in Beast (2). Illustrations of the focal bird species from del Hoyo et al. (1).

|  | **SUPERNOVA** |  |  |  |  |  | **QUAST** |  |  | **BUSCO** |  |  |  |
| --- | --- | --- | --- | --- | --- | --- | --- | --- | --- | --- | --- | --- | --- |
| **Species** | **Assembly length (Mbp)** | **% Missing >10Kb** | **N Scaffolds** | **Scaffold N50 (Kbp)** | **N_Reads (M)** | **Coverage** | **Assembly length (Mbp)** | **N50 (Kbp)** | **Largest_contig (Kpb)** | **%**  **Single-copy** | **% Duplicated** | **% Fragmented** | **% Missing** |
| *Melipotes ater* | 977.4 | 5.1 | 14086 | 20119.6 | 428.8 | 34.0x | 1060.7 | 19472.5 | 87566.3 | 83.5 | 3.3 | 6.6 | 6.6 |
| *Pachycephala schlegelii* | 998.0 | 7.3 | 24119 | 17269.1 | 425.7 | 30.8x | 1143.1 | 13249.4 | 94679.3 | 80.9 | 1.6 | 8.9 | 8.6 |
| *Sericornis nouhuysi* | 1053.5 | 8.0 | 25409 | 19527.2 | 454.3 | 31.1x | 1199.0 | 19112.0 | 56542.6 | 77.6 | 4.2 | 9.6 | 8.6 |
| *Origma robusta* | 966.8 | 9.9 | 28312 | 11142.4 | 425.2 | 31.2x | 1173.7 | 19348.8 | 109299.8 | 76.6 | 1.0 | 13.2 | 9.2 |
| *Peneothello sigillata* | 1013.1 | 8.1 | 27828 | 23793.4 | 403.9 | 35.0x | 1093.6 | 8913.1 | 62227.4 | 78.2 | 1.0 | 10.6 | 10.2 |
| *Ptiloprora guisei* | 967.3 | 7.7 | 22472 | 16587.4 | 381.2 | 34.5x | 1090.8 | 14053.4 | 60309.0 | 80.9 | 1.3 | 8.6 | 9.2 |
| *Rhipidura albolimbata* | 850.3 | 18.1 | 63449 | 78.5 | 433.1 | 35.3x | 1034.0 | 67.1 | 852.8 | 66.7 | 0.0 | 18.1 | 15.2 |
| *Ceyx lepidus* | 869.4 | 8.1 | 20168 | 606.4 | 331.3 | 29.1x | 954.0 | 571.9 | 4695.6 | 74.9 | 1.7 | 10.9 | 12.5 |
| *Thapsinillas afinis* | 941.7 | 11.0 | 31005 | 402.0 | 327.9 | 27.0x | 1051.7 | 358.4 | 3918.6 | 74.6 | 1.3 | 12.5 | 11.6 |
| *Paramythia montium* | 859.6 | 19.8 | 74222 | 44.3 | 305.2 | 23.1x | 1036.4 | 40.6 | 224.3 | 69.3 | 1.0 | 15.5 | 14.2 |
| *Melanocharis versteri* | 799.9 | 27.4 | 87841 | 44.7 | 393.8 | 24.6x | 1014.3 | 40.1 | 167.6 | 67.3 | 0.7 | 19.8 | 12.2 |
| *Ficedula hyperythra* | 815.9 | 24.9 | 87957 | 45.4 | 453.2 | 31.2x | 1017.9 | 40.2 | 156.1 | 72.3 | 1.3 | 14.5 | 11.9 |
| *Toxorhamphus novaeguineae* | 940.8 | 12.7 | 33897 | 1804.5 | 674.4 | 52.9x | 1060.1 | 1621.1 | 18660.3 | 71.3 | 0.3 | 16.8 | 11.6 |
| *Melilestes megarhynchus* | 978.5 | 7.9 | 23361 | 10186.0 | 477.6 | 43.7x | 1088.3 | 9034.2 | 80522.9 | 78.5 | 1.3 | 11.6 | 8.6 |
| *Ifrita kowaldi* | 1005.6 | 5.7 | 19119 | 25436.1 | 579.2 | 55.9x | 1119.9 | 25100.5 | 98025.4 | 81.2 | 0.7 | 9.2 | 8.9 |

**Supplementary Table 1:** **Standard contiguity metrics for all assemblies** using Supernova including total assembly length, number of scaffolds, N50 calculated considering scaffolds, percentage of base assembly missing in scaffolds larger than 10kb, number of reads and coverage. Total assembly length, N50 and maximum scaffold length were also calculated using QUAST. Genome completeness was assessed using BUSCO including percentage of complete single copy, complete duplicated, fragmented and missing BUSCOs.

| ***Ceyx lepidus* (827,972 SNPs)** |  | **_FST_** |
| --- | --- | --- |
| Buru | Seram | 0.15984 |
|  |  |  |
| Buru_1097 | Buru_1435 | 0.0172 |
| Seram_1000 | Seram_1300 | 0.00532 |
|  |  |  |
| Buru_1097 | Seram_1000 | 0.14072 |
| Buru_1097 | Seram_1300 | 0.14227 |
| Buru_1435 | Seram_1000 | 0.13318 |
| Buru_1435 | Seram_1300 | 0.13994 |
|  |  |  |
| ***Ficedula buruensis* (966,589 SNPs)** |  | **F_ST_** |
| Buru | Seram | 0.12748 |
|  |  |  |
| Buru_1097 | Buru_1435 | 0.0024587 |
|  |  |  |
| Buru_1097 | Seram_1000 | 0.11551 |
| Buru_1435 | Seram_1000 | 0.11403 |
|  |  |  |
| ***Ficedula hyperythra* (1,607,232 SNPs)** |  | **F_ST_** |
| Buru | Seram | 0.039183 |
|  |  |  |
| ***Pachycephala macrorhyncha* (3,555,034 SNPs)** |  | **F_ST_** |
| Buru | Seram | 0.087418 |
|  |  |  |
| Buru_1097 | Buru_1435 | -0.00048775 |
| Seram_1000 | Seram_1300 | 0.001694 |
|  |  |  |
| Buru_1097 | Seram_1000 | 0.073181 |
| Buru_1097 | Seram_1300 | 0.081187 |
| Buru_1435 | Seram_1000 | 0.072061 |
| Buru_1435 | Seram_1300 | 0.080301 |
|  |  |  |
| ***Thapsinillas affinis* (817,005 SNPs)** |  | **F_ST_** |
| Buru | Seram | 0.14511 |
|  |  |  |
| ***Melipotes fumigatus/ater* (1,490,622 SNPs)** |  | **F_ST_** |
| Wilhelm | Huon | 0.08238 |
|  |  |  |
| Wilhelm_1700 | Wilhelm_3200+3700 | 0.0029554 |
|  |  |  |
| ***Ifrita kowaldi* (1,007,491 SNPs)** |  | **F_ST_** |
| Wilhelm | Huon | 0.072839 |
|  |  |  |
| Huon_2300 | Huon_2950 | 0.034225 |
|  |  |  |
| Wilhelm | Huon_2300 | 0.044393 |
| Wilhelm | Huon_2950 | 0.081431 |
|  |  |  |
| ***Melanocharis versteri* (1,414,634 SNPs)** |  | **F_ST_** |
| Wilhelm | Huon | 0.012366 |
|  |  |  |
| ***Origma robusta* (3,555,571 SNPs)** |  | **F_ST_** |
| Wilhelm | Huon | 0.02258 |
|  |  |  |
| Wilhelm_1700 | Wilhelm_2700 | 0.0057128 |
| Wilhelm_1700 | Wilhelm_3700 | 0.011681 |
| Wilhelm_2700 | Wilhelm_3700 | 0.0055156 |
|  |  |  |
| Wilhelm_1700 | Huon | 0.017878 |
| Wilhelm_2700 | Huon | 0.016152 |
| Wilhelm_3700 | Huon | 0.01776 |
|  |  |  |
| ***Ptiloprora guisei* (11,840,599 SNPs)** |  | **F_ST_** |
| Wilhelm | Huon | 0.027875 |
|  |  |  |
| ***Rhipidura albolimbata* (3,322,692 SNPs)** |  | **F_ST_** |
| Wilhelm | Huon | 0.010875 |
|  |  |  |
| Wilhelm_1700 | Wilhelm_2700 | 0.0030105 |
|  |  |  |
| ***Melilestes megarhynchus* (4,759,364 SNPs)** |  | **F_ST_** |
| Wilhelm | Huon | 0.0017461 |
|  |  |  |
| Wilhelm | Lowlands | -0.00455 |
| Huon | Lowlands | 0.0040077 |
|  |  |  |
| ***Toxorhamphus novaeguineae* (10,039,403 SNPs)** |  | **F_ST_** |
| Wilhelm | Huon | 0.0015122 |
|  |  |  |
| Wilhelm | Lowlands | 0.00032116 |
| Huon | Lowlands | 0.00017707 |
|  |  |  |
| ***Pachyephala schlegelii* (1,992,013 SNPs)** |  | **F_ST_** |
| Wilhelm | Huon | 0.037053 |
| Wilhelm | Scratchley | 0.013103 |
| Huon | Scratchley | 0.046222 |
|  |  |  |
| ***Paramythia montium* (1,418,592 SNPs)** |  | **F_ST_** |
| Wilhelm | Huon | 0.09274 |
| Wilhelm | Scratchley | 0.099558 |
| Huon | Scratchley | 0.1029 |
|  |  |  |
| Wilhelm_2700 | Wilhelm_3700 | 0.0023143 |
|  |  |  |
| Wilhelm_2700 | Huon | 0.092442 |
| Wilhelm_3700 | Huon | 0.092249 |
| Wilhelm_2700 | Scratchley | 0.095104 |
| Wilhelm_3700 | Scratchley | 0.096624 |
|  |  |  |
| ***Peneothello sigillata* (3,344,876 SNPs)** |  | **F_ST_** |
| Wilhelm | Huon | 0.051377 |
| Wilhelm | Scratchley | 0.058752 |
| Huon | Scratchley | 0.073254 |
|  |  |  |
| ***Sericornis nouhuysi* (6,127,744 SNPs)** |  | **F_ST_** |
| Wilhelm | Huon | 0.034097 |
| Wilhelm | Scratchley | 0.0019496 |
| Huon | Scratchley | 0.0024803 |
|  |  |  |
| Wilhelm_1700 | Wilhelm_2700+3200 | 0.014914 |
|  |  |  |
| ***Aethomyias papuensis* (4,095,422 SNPs)** |  | **F_ST_** |
| Wilhelm | Huon | 0.012793 |
| Wilhelm | Scratchley | -0.0022371 |
| Huon | Scratchley | 0.019918 |

**Supplementary Table 2:** **Summary of genetic differentiation (F_ST_) between populations.** When possible, genetic differentiation between different elevations was also calculated.

| **Species** | **SciLife Code** | **Date** | **Locality** | **Elevation (m)** | **GPS coordinate** | **Sex** | **Sample type** | **Voucher Number** |
| --- | --- | --- | --- | --- | --- | --- | --- | --- |
| *Melipotes ater* | P10712_101 | 11/09/2016 | Papua New Guinea:Wasaunon, Huon | 2950 | 6.095266667 S 146.9157 E | Unknown | Blood | NHMD 615241 |
| *Pachycephala schlegelii* | P10712_102 | 13/09/2016 | Papua New Guinea:Wasaunon, Huon | 2950 | 6.095266667 S 146.9157 E | Unknown | Blood | NHMD 615259 |
| *Sericornis nouhuysi* | P10712_103 | 11/09/2016 | Papua New Guinea:Wasaunon, Huon | 2950 | 6.095266667 S 146.9157 E | Unknown | Blood | NHMD 615417 |
| *Origma robusta* | P10908_101 | 10/09/2016 | Papua New Guinea:Wasaunon, Huon | 2950 | 6.095266667 S 146.9157 E | Unknown | Blood | NHMD 615219 |
| *Peneothello sigillata* | P10908_102 | 10/09/2016 | Papua New Guinea:Wasaunon, Huon | 2950 | 6.095266667 S 146.9157 E | Unknown | Blood | NHMD 615286 |
| *Ptiloprora guisei* | P10908_103 | 10/09/2016 | Papua New Guinea:Wasaunon, Huon | 2950 | 6.095266667 S 146.9157 E | Unknown | Blood | NHMD 615332 |
| *Rhipidura albolimbata* | P10908_104 | 29/09/2015 | Papua New Guinea:Bruno Sawmill, Mt Wilhelm | 2700 | 5.815833333 S 145.150556 E | Unknown | Muscle | NHMD 164070 |
| *Ceyx lepidus* | P10908_105 | 10/02/2012 | Indonesia:Seram, above Kanikeh, Camp 1 | 1000 | 3.1385 S 129.4823667 E | Male | Muscle | NHMD 131364 |
| *Thapsinillas afinis* | P10908_106 | 03/02/2011 | Indonesia:Buru,ESE of Waikega, Camp 2 | 693 | 3.206666667 S 126.0758333 E | Female | Muscle | MZB 32431 |
| *Paramythia montium* | P12064_103 | 24/09/2015 | Papua New Guinea:Lake Aunde, Mt Wilhelm | 3700 | 5.786111111 S 145.0588889 E | Female | Muscle | NHMD 138733 |
| *Melanocharis versteri* | P12064_104 | 29/09/2015 | Papua New Guinea:Bruno Sawmill, Mt Wilhelm | 2700 | 5.815833333 S 145.150556 E | Unknown | Muscle | NHMD 163736 |
| *Ficedula hyperythra* | P12064_105 | 10/02/2012 | Indonesia:Seram, above Kanikeh, Camp 2 | 1300 | 3.145 S 129.474E | Unknown | Muscle | MZB 33228 |
| *Toxorhamphus novaeguineae* | P12853_101 | 18/10/2015 | Papua New Guinea:Kausi, Mt Wilhelm | 200 | 5.7425 S 145.3336111 E | Unknown | Muscle | NHMD 138808 |
| *Melilestes megarhynchus* | P12853_102 | 22/08/2017 | Papua New Guinea:Wanang Village, Lowlands | 200 | 5.228266667 S 145.1731333 E | Unknown | Blood | NHMD 615658 |
| *Ifrita kowaldi* | P12853_103 | 14/09/2016 | Papua New Guinea:Wasaunon, Huon | 2950 | 6.095266667 S 146.9157 E | Unknown | Blood | NHMD 217730 |

**Supplementary Table 3:** **Details of individuals used for *de novo* genome sequencing** including the date of collection, the geographical location, elevation, GPS coordinate, sex, tissue type and voucher number.

**Supplementary Methods.** Code used to test a range of models in fastsimcoal2.

**1) NULL MODEL**

[PARAMETERS]

//#isInt? #name #dist.#min #max

//all Ns are in number of haploid individuals

1 N1 unif 10 1e6 output

1 N2 unif 10 1e6 output

1 NANC unif 10 1e6 output

1 TDIV unif 100 1e6 output

[RULES]

[COMPLEX PARAMETERS]

0 R1 = NANC/N1 hide

//Number of population samples (demes)

2

//Population effective sizes (number of genes)

N1

N2

//Sample sizes

10

10

//Growth rates : negative growth implies population expansion

0

0

//Number of migration matrices : 0 implies no migration between demes

0

//historical event: time, source, sink, migrants, new size, new growth rate, migr. matrix

2 historical event

TDIV 0 0 0 R1 0 0

TDIV 1 0 1 1 0 0

//Number of independent loci [chromosome]

1 0

//Per chromosome: Number of contiguous linkage Block: a block is a set of contiguous loci

1

//per Block:data type, number of loci, per generation recombination and mutation rates and optional parameters

FREQ 1 0 3e-9 OUTEXP

**2) MIGRATION**

[PARAMETERS]

//#isInt? #name #dist.#min #max

//all Ns are in number of haploid individuals

1 N1 unif 10 1e6 output

1 N2 unif 10 1e6 output

1 NANC unif 10 1e6 output

1 TDIV unif 100 1e6 output

0 M12 logunif 1e-20 1e-1 output

0 M21 logunif 1e-20 1e-1 output

[RULES]

[COMPLEX PARAMETERS]

0 R1 = NANC/N1 hide

//Number of population samples (demes)

2

//Population effective sizes (number of genes)

N1

N2

//Sample sizes

10

10

//Growth rates : negative growth implies population expansion

0

0

//Number of migration matrices : 0 implies no migration between demes

2

//Migration matrix

0 M12

M21 0

//Migration matrix

0 0

0 0

//historical event: time, source, sink, migrants, new size, new growth rate, migr. matrix

2 historical event

TDIV 0 0 0 R1 0 1

TDIV 1 0 1 1 0 1

//Number of independent loci [chromosome]

1 0

//Per chromosome: Number of contiguous linkage Block: a block is a set of contiguous loci

1

//per Block:data type, number of loci, per generation recombination and mutation rates and optional parameters

FREQ 1 0 3e-9 OUTEXP

**3) EXPONENTIAL GROWTH**

[PARAMETERS]

//#isInt? #name #dist.#min #max

//all Ns are in number of haploid individuals

1 N1 unif 10 1e6 output

1 N2 unif 10 1e6 output

1 NANC unif 10 1e6 output

1 TDIV unif 100 1e6 output

0 RATE1 unif -0.0001 0.0001 output

0 RATE2 unif -0.0001 0.0001 output

[RULES]

[COMPLEX PARAMETERS]

0 R1 = NANC/N1 hide

//Number of population samples (demes)

2

//Population effective sizes (number of genes)

N1

N2

//Sample sizes

10

10

//Growth rates : negative growth implies population expansion

RATE1

RATE2

//Number of migration matrices : 0 implies no migration between demes

0

//historical event: time, source, sink, migrants, new size, new growth rate, migr. matrix

2 historical event

TDIV 0 0 1 R1 0 0

TDIV 1 0 1 1 0 0

//Number of independent loci [chromosome]

1 0

//Per chromosome: Number of contiguous linkage Block: a block is a set of contiguous loci

1

//per Block:data type, number of loci, per generation recombination and mutation rates and optional parameters

FREQ 1 0 3e-9 OUTEXP

**4) EXPONENTIAL GROWTH + MIGRATION**

[PARAMETERS]

//#isInt? #name #dist.#min #max

//all Ns are in number of haploid individuals

1 N1 unif 10 1e6 output

1 N2 unif 10 1e6 output

1 NANC unif 10 1e6 output

1 TDIV unif 100 1e6 output

0 RATE1 unif -0.0001 0.0001 output

0 RATE2 unif -0.0001 0.0001 output

0 M12 logunif 1e-20 1e-1 output

0 M21 logunif 1e-20 1e-1 output

[RULES]

[COMPLEX PARAMETERS]

0 R1 = NANC/N1 hide

//Number of population samples (demes)

2

//Population effective sizes (number of genes)

N1

N2

//Sample sizes

10

10

//Growth rates : negative growth implies population expansion

RATE1

RATE2

//Number of migration matrices : 0 implies no migration between demes

2

//Migration matrix

0 M12

M21 0

//Migration matrix

0 0

0 0

//historical event: time, source, sink, migrants, new size, new growth rate, migr. matrix

2 historical event

TDIV 0 0 1 R1 0 1

TDIV 1 0 1 1 0 1

//Number of independent loci [chromosome]

1 0

//Per chromosome: Number of contiguous linkage Block: a block is a set of contiguous loci

1

//per Block:data type, number of loci, per generation recombination and mutation rates and optional parameters

FREQ 1 0 3e-9 OUTEXP

**5) BOTTLENECK**

[PARAMETERS]

//#isInt? #name #dist.#min #max

//all Ns are in number of haploid individuals

1 N1 unif 10 1e6 output

1 N2 unif 10 1e6 output

1 NANC unif 10 1e6 output

1 NINT unif 10 1e6 output

1 TDIV unif 100 1e6 output

1 TSUD unif 100 1e6 output

[RULES]

NINT > N1

NINT > N2

TDIV > TSUD

[COMPLEX PARAMETERS]

0 R1 = NANC/NINT hide

0 R2 = NINT/N1 hide

//Number of population samples (demes)

2

//Population effective sizes (number of genes)

N1

N2

//Sample sizes

10

10

//Growth rates : negative growth implies population expansion

0

0

//Number of migration matrices : 0 implies no migration between demes

0

//historical event: time, source, sink, migrants, new size, new growth rate, migr. matrix

3 historical event

TSUD 0 0 1 R2 0 0

TDIV 0 0 1 R1 0 0

TDIV 1 0 1 1 0 0

//Number of independent loci [chromosome]

1 0

//Per chromosome: Number of contiguous linkage Block: a block is a set of contiguous loci

1

//per Block:data type, number of loci, per generation recombination and mutation rates and optional parameters

FREQ 1 0 3e-9 OUTEXP

**6) BOTTLENECK+MIGRATION**

[PARAMETERS]

//#isInt? #name #dist.#min #max

//all Ns are in number of haploid individuals

1 N1 unif 10 1e6 output

1 N2 unif 10 1e6 output

1 NANC unif 10 1e6 output

1 NINT unif 10 1e6 output

1 TDIV unif 100 1e6 output

1 TSUD unif 100 1e6 output

0 M12 logunif 1e-20 1e-1 output

0 M21 logunif 1e-20 1e-1 output

[RULES]

NINT > N1

NINT > N2

TDIV > TSUD

[COMPLEX PARAMETERS]

0 R1 = NANC/NINT hide

0 R2 = NINT/N1 hide

//Number of population samples (demes)

2

//Population effective sizes (number of genes)

N1

N2

//Sample sizes

10

10

//Growth rates : negative growth implies population expansion

0

0

//Number of migration matrices : 0 implies no migration between demes

2

//Migration matrix

0 M12

M21 0

//Migration matrix

0 0

0 0

//historical event: time, source, sink, migrants, new size, new growth rate, migr. matrix

3 historical event

TSUD 0 0 1 R2 0 1

TDIV 0 0 1 R1 0 1

TDIV 1 0 1 1 0 1

//Number of independent loci [chromosome]

1 0

//Per chromosome: Number of contiguous linkage Block: a block is a set of contiguous loci

1

//per Block:data type, number of loci, per generation recombination and mutation rates and optional parameters

FREQ 1 0 3e-9 OUTEXP

**7) SUDDEN EXPANSION**

[PARAMETERS]

//#isInt? #name #dist.#min #max

//all Ns are in number of haploid individuals

1 N1 unif 10 1e6 output

1 N2 unif 10 1e6 output

1 NANC unif 10 1e6 output

1 NINT unif 10 1e6 output

1 TDIV unif 100 1e6 output

1 TSUD unif 100 1e6 output

[RULES]

N1 > NINT

N2 > NINT

TDIV > TSUD

[COMPLEX PARAMETERS]

0 R1 = NANC/NINT hide

0 R2 = NINT/N1 hide

//Number of population samples (demes)

2

//Population effective sizes (number of genes)

N1

N2

//Sample sizes

10

10

//Growth rates : negative growth implies population expansion

0

0

//Number of migration matrices : 0 implies no migration between demes

0

//historical event: time, source, sink, migrants, new size, new growth rate, migr. matrix

3 historical event

TSUD 0 0 1 R2 0 0

TDIV 0 0 1 R1 0 0

TDIV 1 0 1 1 0 0

//Number of independent loci [chromosome]

1 0

//Per chromosome: Number of contiguous linkage Block: a block is a set of contiguous loci

1

//per Block:data type, number of loci, per generation recombination and mutation rates and optional parameters

FREQ 1 0 3e-9 OUTEXP

**8) SUDDEN EXPANSION+MIGRATION**

[PARAMETERS]

//#isInt? #name #dist.#min #max

//all Ns are in number of haploid individuals

1 N1 unif 10 1e6 output

1 N2 unif 10 1e6 output

1 NANC unif 10 1e6 output

1 NINT unif 10 1e6 output

1 TDIV unif 100 1e6 output

1 TSUD unif 100 1e6 output

0 M12 logunif 1e-20 1e-1 output

0 M21 logunif 1e-20 1e-1 output

[RULES]

N1 > NINT

N2 > NINT

TDIV > TSUD

[COMPLEX PARAMETERS]

0 R1 = NANC/NINT hide

0 R2 = NINT/N1 hide

//Number of population samples (demes)

2

//Population effective sizes (number of genes)

N1

N2

//Sample sizes

10

10

//Growth rates : negative growth implies population expansion

0

0

//Number of migration matrices : 0 implies no migration between demes

2

//Migration matrix

0 M12

M21 0

//Migration matrix

0 0

0 0

//historical event: time, source, sink, migrants, new size, new growth rate, migr. matrix

3 historical event

TSUD 0 0 1 R2 0 1

TDIV 0 0 1 R1 0 1

TDIV 1 0 1 1 0 1

//Number of independent loci [chromosome]

1 0

//Per chromosome: Number of contiguous linkage Block: a block is a set of contiguous loci

1

//per Block:data type, number of loci, per generation recombination and mutation rates and optional parameters

FREQ 1 0 3e-9 OUTEXP

**Supplementary References**

1. J. del Hoyo, A. Elliott, J. Sargatal, D. A. Christie, E. de Juana, (Eds.) Handbook of the Birds of the World Alive. Lynx Edicions, Barcelona. (retrieved from https://www.hbw.com on 24/09/2019).

2. A. J. Drummond, M. A. Suchard, D. Xie, A. Rambaut, Bayesian phylogenetics with BEAUti and the BEAST 1.7. *Mol. Biol. Evol.* **29,** 1969–1973 (2012).
