# Supplementary material for: The formation of avian montane diversity across barriers and along elevational gradients
Source: Nat Commun. 2022 Jan 12;13:268. doi: 10.1038/s41467-021-27858-5 (PMC8755808; doi:10.1038/s41467-021-27858-5)
Supplement: Supplementary file 5 — Reporting Summary [file 41467_2021_27858_MOESM5_ESM.pdf]

nature research

Corresponding author(s): Knud Andreas Jønsson

Last updated by author(s): 2 December 2021

Reporting Summary

Nature Research wishes to improve the reproducibility of the work that we publish. This form provides structure for consistency and transparency in reporting. For further information on Nature Research policies, see Authors & Referees and the Editorial Policy Checklist.

Statistics

For all statistical analyses, confirm that the following items are present in the figure legend, table legend, main text, or Methods section.

n/a	Confirmed	
☐	☑	The exact sample size (*n*) for each experimental group/condition, given as a discrete number and unit of measurement
☐	☑	A statement on whether measurements were taken from distinct samples or whether the same sample was measured repeatedly
☐	☑	The statistical test(s) used AND whether they are one- or two-sided<br>*Only common tests should be described solely by name; describe more complex techniques in the Methods section.*
☐	☑	A description of all covariates tested
☐	☑	A description of any assumptions or corrections, such as tests of normality and adjustment for multiple comparisons
☐	☑	A full description of the statistical parameters including central tendency (e.g. means) or other basic estimates (e.g. regression coefficient) AND variation (e.g. standard deviation) or associated estimates of uncertainty (e.g. confidence intervals)
☑	☐	For null hypothesis testing, the test statistic (e.g. *F*, *t*, *r*) with confidence intervals, effect sizes, degrees of freedom and *P* value noted<br>*Give P values as exact values whenever suitable.*
☑	☐	For Bayesian analysis, information on the choice of priors and Markov chain Monte Carlo settings
☑	☐	For hierarchical and complex designs, identification of the appropriate level for tests and full reporting of outcomes
☑	☐	Estimates of effect sizes (e.g. Cohen's *d*, Pearson's *r*), indicating how they were calculated

Our web collection on statistics for biologists contains articles on many of the points above.

Software and code

Policy information about availability of computer code

Data collection	Fastsimcoal code used herein is provided in Supplementary Methods.<br>AFS generated using the scripts in https://gitbub.com/shenglin-liu/vcf2sfs
Data analysis	Supernova v2.0.1; BUSCO v4.0.2; QUAST v5.0.1; FASTQC v0.11.9; FASTX v0.0.14; Trimmomatic v0.36; BWA v0.91; BCFtools v1.9; vcftools v0.1.14; PLINK v1.90b3.42; PCAngsd v0.95; STRUCTURE v2.3.4, PSMC v0.6.5; Fastsimcoal 2 fsc26, Princomp in R

For manuscripts utilizing custom algorithms or software that are central to the research but not yet described in published literature, software must be made available to editors/reviewers. We strongly encourage code deposition in a community repository (e.g. GitHub). See the Nature Research guidelines for submitting code & software for further information.

Data

Policy information about availability of data

All manuscripts must include a data availability statement. This statement should provide the following information, where applicable:

- Accession codes, unique identifiers, or web links for publicly available datasets
- A list of figures that have associated raw data
- A description of any restrictions on data availability

All sequencing data have been deposited under the BioProject accession number PRJNA637995 and PRJNA580350

Field-specific reporting

Please select the one below that is the best fit for your research. If you are not sure, read the appropriate sections before making your selection.

☐ Life sciences ☐ Behavioural & social sciences ☑ Ecological, evolutionary & environmental sciences

For a reference copy of the document with all sections, see nature.com/documents/nr-reporting-summary-flat.pdf

Life sciences study design

All studies must disclose on these points even when the disclosure is negative.

Sample size

Data exclusions

Replication

Randomization

Blinding

Behavioural & social sciences study design

All studies must disclose on these points even when the disclosure is negative.

Study description

Research sample

Sampling strategy

Data collection

Timing

Data exclusions

Non-participation

Randomization

Ecological, evolutionary & environmental sciences study design

All studies must disclose on these points even when the disclosure is negative.

Study description: Comparative study of avian montane diversity using de novo genome sequencing and assembly of bird species pairs from the Indo-Pacific.

Research sample: Details on all denovo genomes and all resequenced individuals including species, location, collection date, coordinates, altitude and sex are summarized in Tables S1 and S2.

Sampling strategy: We resequenced 226 individuals from 18 bird species, ranging from 9 to 18 individuals per species, to ensure sufficient numbers for statistic analysis. The species were chosen to represent a broad selection of elevations.

Data collection: Birds were collected using mist nets, which is the usual method for collecting birds in the wild. Pen and paper was used to note details. Birds were collected by K.A.Jønsson and collaborators.

Timing and spatial scale: Samples were obtained from Papua New Guinea and Indonesia (islands of Buru and Seram) between 2011-2018. Samples were obtained over several years as it was not possible to gather all samples in 1 year.

Data exclusions: We excluded from the study 2 individuals identified as half-siblings using kinship anaysis. Sampling of closely related individuals can dramatically bias estimates of population structure and demographics.

Reproducibility: All methods are thoroughly described to allow reproducibility.

Randomization: N/A. All individuals sampled were included in the analysis and no randomization was involved.

Blinding: N/A. All individuals sampled were included in the analysis.

Did the study involve field work? ☑ Yes ☐ No

Field work, collection and transport

Field conditions: Climatic conditions during sampling was not recorded but no environmental conditions were relevant to the study

Location: Islands of Buru and Seram in Indonesia and Papua New Guinea

Access and import/export	This research complies with all relevant ethical regulations in Indonesia and in Papua New Guinea. Research in New Guinea was approved by the State Ministry of Research and Technology (RISTEK, permits SURAT IZIN PENELITIAN: 013/SIP/FRP/I/2011 and 026/SIP/FRP/I/2012). The PNG National Museum and art gallery and the Conservation and Environment Protection Authority (CEPA) of Papua New Guinea provided research permits and export permits. Research in Indonesia was approved by the State Ministry of Research and Technology (RISTEK, permits SURAT IZIN PENELITIAN: 013/SIP/FRP/I/2011 and 026/SIP/FRP/I/2012), the Ministry of Forestry (Republic of Indonesia), the Research Center for Biology, Indonesian Institute of Sciences (RCB-LIPI) and the Bogor Zoological Museum. Collectively, these institutions provided permits to carry out fieldwork in Indonesia and to export select samples.
Disturbance	Mistnets were checked regularly (at least every 30 minutes) and a maximum of 10 individuals per bird population were collected.

Reporting for specific materials, systems and methods

We require information from authors about some types of materials, experimental systems and methods used in many studies. Here, indicate whether each material, system or method listed is relevant to your study. If you are not sure if a list item applies to your research, read the appropriate section before selecting a response.

Materials & experimental systems

n/a	Involved in the study
☑	☐ Antibodies
☑	☐ Eukaryotic cell lines
☑	☐ Palaeontology
☐	☑ Animals and other organisms
☑	☐ Human research participants
☑	☐ Clinical data

Methods

n/a	Involved in the study
☑	☐ ChIP-seq
☑	☐ Flow cytometry
☑	☐ MRI-based neuroimaging

Antibodies

Antibodies used	NA
Validation	NA

Eukaryotic cell lines

Policy information about cell lines

Cell line source(s)	NA
Authentication	NA
Mycoplasma contamination	NA
Commonly misidentified lines (See ICLAC register)	NA

Palaeontology

Specimen provenance	NA

Specimen deposition

NA

Dating methods

NA

☐ Tick this box to confirm that the raw and calibrated dates are available in the paper or in Supplementary Information.

Animals and other organisms

Policy information about studies involving animals; ARRIVE guidelines recommended for reporting animal research

Laboratory animals

No laboratory animals were used

Wild animals

Birds were collected in the field using nets and killed to obtain samples for genetic studies. Birds were killed in a humane way by cervical dislocation to ensure that the animals did not suffer unnecessarily..

Field-collected samples

No live birds were transported.

Ethics oversight

This research complies with all relevant ethical regulations in Indonesia and in Papua New Guinea. Research in New Guinea was approved by the State Ministry of Research and Technology (RISTEK, permits SURAT IZIN PENELITIAN: 013/SIP/FRP/I/2011 and 026/SIP/FRP/I/2012). The PNG National Museum and art gallery and the Conservation and Environment Protection Authority (CEPA) of Papua New Guinea provided research permits and export permits. Research in Indonesia was approved by the State Ministry of Research and Technology (RISTEK, permits SURAT IZIN PENELITIAN: 013/SIP/FRP/I/2011 and 026/SIP/FRP/I/2012), the Ministry of Forestry (Republic of Indonesia), the Research Center for Biology, Indonesian Institute of Sciences (RCB-LIPI) and the Bogor Zoological Museum. Collectively, these institutions provided permits to carry out fieldwork in Indonesia and to export select samples.

Note that full information on the approval of the study protocol must also be provided in the manuscript.

Human research participants

Policy information about studies involving human research participants

Population characteristics

NA

Recruitment

NA

Ethics oversight

NA

Note that full information on the approval of the study protocol must also be provided in the manuscript.

Clinical data

Policy information about clinical studies

All manuscripts should comply with the ICMJE guidelines for publication of clinical research and a completed CONSORT checklist must be included with all submissions.

Clinical trial registration

NA

Study protocol

NA

Data collection

NA

Outcomes

NA

ChIP-seq

Data deposition

- [ ] Confirm that both raw and final processed data have been deposited in a public database such as GEO.
- [ ] Confirm that you have deposited or provided access to graph files (e.g. BED files) for the called peaks.

Data access links
May remain private before publication.

NA

Files in database submission

NA

Genome browser session
(e.g. UCSC)

NA

Methodology

Replicates

NA

Sequencing depth

NA

Antibodies

NA

Peak calling parameters

NA

Data quality

NA

Software

NA

Flow Cytometry

Plots

Confirm that:

- [ ] The axis labels state the marker and fluorochrome used (e.g. CD4-FITC).
- [ ] The axis scales are clearly visible. Include numbers along axes only for bottom left plot of group (a 'group' is an analysis of identical markers).
- [ ] All plots are contour plots with outliers or pseudocolor plots.
- [ ] A numerical value for number of cells or percentage (with statistics) is provided.

Methodology

Sample preparation	NA
Instrument	NA
Software	NA
Cell population abundance	NA
Gating strategy	NA

- [ ] Tick this box to confirm that a figure exemplifying the gating strategy is provided in the Supplementary Information.

Magnetic resonance imaging

Experimental design

Design type	NA
Design specifications	NA
Behavioral performance measures	NA

Acquisition

Imaging type(s)

NA

Field strength

NA

Sequence & imaging parameters

NA

Area of acquisition

NA

Diffusion MRI ☐ Used ☑ Not used

Preprocessing

Preprocessing software

NA

Normalization

NA

Normalization template

NA

Noise and artifact removal

NA

Volume censoring

NA

Statistical modeling & inference

Model type and settings

NA

Effect(s) tested

NA

Specify type of analysis: ☐ Whole brain ☐ ROI-based ☐ Both

Statistic type for inference
(See Eklund et al. 2016)

NA

Correction

NA

Models & analysis

n/a	Involved in the study
☑	☐ Functional and/or effective connectivity
☑	☐ Graph analysis
☑	☐ Multivariate modeling or predictive analysis

Functional and/or effective connectivity	NA
Graph analysis	NA
Multivariate modeling and predictive analysis	NA
